# Thymol-loaded PLGA nanoparticles: an efficient approach for acne treatment

**DOI:** 10.1186/s12951-021-01092-z

**Published:** 2021-11-08

**Authors:** Camila Folle, Ana M. Marqués, Natalia Díaz-Garrido, Marta Espina, Elena Sánchez-López, Josefa Badia, Laura Baldoma, Ana Cristina Calpena, Maria Luisa García

**Affiliations:** 1 Department of Pharmacy and Pharmaceutical Technology and Physical Chemistry, Faculty of Pharmacy and Food Sciences, University of Barcelona, 08028 Barcelona, Spain; 2 Department of Biology, Healthcare and Environment, Faculty of Pharmacy and Food Sciences, University of Barcelona, 08028 Barcelona, Spain; 3 Department of Biochemistry and Physiology, Biochemistry and Biomolecular Science, University of Barcelona, 08028 Barcelona, Spain; 4 Institute of Biomedicine of the University of Barcelona (IBUB), 08028 Barcelona, Spain; 5 Research Institute Sant Joan De Déu (IR-SJD), 08950 Barcelona, Spain; 6 Institute of Nanoscience and Nanotechnology (IN2UB), University of Barcelona, 08028 Barcelona, Spain

**Keywords:** Acne, Thymol, PLGA nanoparticles, Skin delivery system, *Cutibacterium acnes*, Skin microbiota, Antimicrobial, Antioxidant

## Abstract

**Background:**

Acne is a common skin disorder that involves an infection inside the hair follicle, which is usually treated with antibiotics, resulting in unbalanced skin microbiota and microbial resistance. For this reason, we developed polymeric nanoparticles encapsulating thymol, a natural active compound with antimicrobial and antioxidant properties. In this work, optimization physicochemical characterization, biopharmaceutical behavior and therapeutic efficacy of this novel nanostructured system were assessed.

**Results:**

Thymol NPs (TH-NP) resulted on suitable average particle size below 200 nm with a surface charge around − 28 mV and high encapsulation efficiency (80%). TH-NP released TH in a sustained manner and provide a slow-rate penetration into the hair follicle, being highly retained inside the skin. TH-NP possess a potent antimicrobial activity against *Cutibacterium acnes* and minor effect towards *Staphylococcus epidermis*, the major resident of the healthy skin microbiota. Additionally, the stability and sterility of developed NPs were maintained along storage.

**Conclusion:**

TH-NP showed a promising and efficient alternative for the treatment of skin acne infection, avoiding antibiotic administration, reducing side effects, and preventing microbial drug resistance, without altering the healthy skin microbiota. Additionally, TH-NP enhanced TH antioxidant activity, constituting a natural, preservative-free, approach for acne treatment.

**Graphical Abstract:**

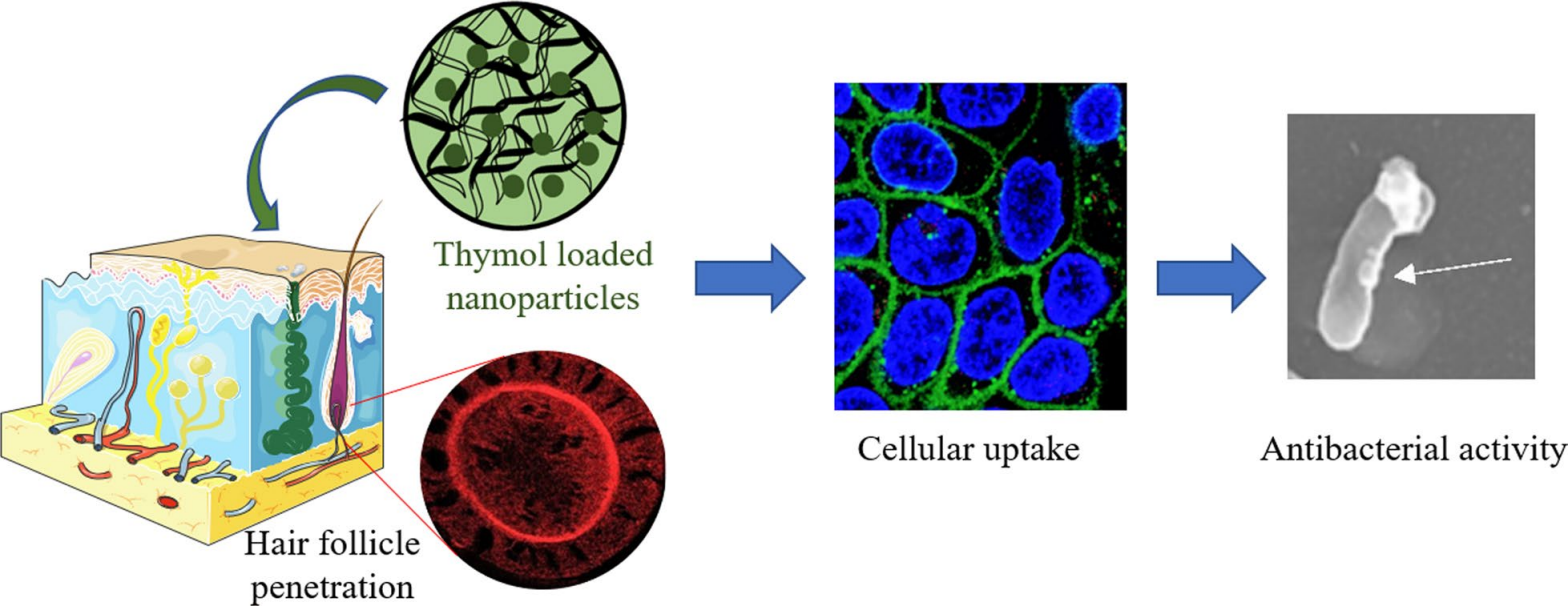

**Supplementary Information:**

The online version contains supplementary material available at 10.1186/s12951-021-01092-z.

## Background

Acne is a common skin disorder, known as *Acne vulgaris*, that affects a large number of the population. Several factors, such as hormones, diet, stress and environmental pollution, among others, may contribute to acne development. These factors trigger hyperactivity of sebaceous glands that produce elevated levels of sebum, hyperkeratosis by blockage of the hair follicle and, additionally, contribute to the excessive microbiota reproduction [1].

The skin is divided into three functional layers that surround the hair follicle. The base is found on the dermis–hypodermis junction, just above the fat tissue. Production of sebum occurs in the sebaceous gland, which is located inside the dermis. The upper layers of the epidermis are composed by keratinocyte cells surrounding the hair top-to-bottom. The external skin layer, stratum corneum (SC), protects the skin from the external environment, being considered the main challenge for several topical drugs penetration.

Several microorganisms reside in the skin and can be classified as resident or transient microbiota. They are normally gram-positive bacteria, not regarded as pathogens, which survive longer on intact skin than gram-negative transient species. The protective microbiota functions are believed to confer microbial antagonism activity and nutrient competition for the stability of the dermal ecosystem, preventing the adherence of pathogens and maintaining the skin health balanced [2]. *Staphylococcus epidermidis* is the most abundant colonizer of human skin and, despite considered a benign microorganism, it is highly present in acne lesions [3]. It is ubiquitously found mainly concentrated on the upper layers of the skin and may have a probiotic function by preventing colonization of pathogenic bacteria such as *Staphylococcus aureus* [4]. Concerning *Cutibacterium acnes*, previously known as *Propionibacterium acnes*, it is a resident bacteria, mainly located surrounding the hair follicle, which is likely to proliferate under unbalanced function of the sebaceous glands, leading to acne development, swelling and inflammation [5]. Hence, *C. acnes* has a dual activity on the skin microbiota, being a non-pathogen essential for sebum control, as well as an active pathogen on acne infection and inflammation. Additionally, it maintains the acidic pH of the pilosebaceous follicle by hydrolyzing sebum triglycerides and via propionic acid secretion. Moreover, it has been previously stated that acne might be the result of an unbalanced equilibrium between *C. acnes* and *S. epidermidis* [6]. Therefore, a suitable treatment for acne should provide good antimicrobial activity but acquiring mild effect to the healthy microbiota.

Nanoparticles (NP) are a good approach to enter the hair follicle and release the antimicrobial agent directly in the acne lesion [7, 8]. The fat deposit on the subcutaneous tissue may behave as a deep compartment for the drug, delaying its entry in the blood circulation [9]. Polymeric nanospheres of poly(lactic-co-glycolic) acid (PLGA), form a matrix structure containing the entrapped active compound. PLGA, approved by the Food and Drug Administration (FDA), is known to be safe for dermal applications due to its bioavailability and biodegradable profile. One of the advantages of PLGA NPs entrapping highly volatile compounds is that their production can be performed at room temperature. Moreover, nanotechnology also has several advantages in dermal drug delivery since small particle diameters tend to penetrate into the deep skin (DS), withdraw the drug in a controlled manner and be mainly retained in the deeper layers [8, 10]. The size and flexibility of PLGA NPs enable entry into host cells via endocytosis, thus allowing intracellular release. In addition, they are easily phagocytosed by host phagocytes [11].

In this area, several clinical assays highlight the successful combination of nutraceuticals/cosmetic compounds and their encapsulation in nanostructured systems for topical acne treatment. In this area, Abd-Allah and colleagues showed successful reduction of acne inflammatory lesions with nicotinamide loaded chitosan nanoparticles [12]. Furthermore, quercetin has also been encapsulated into nanovesicles and proven to be effective both as antibacterial and anti-inflammatory [13].

Among the wide range of nutraceutical compounds, thymol (TH), is a multifunctional monoterpene of aromatic phenolic structure, with a volatile compound with a strong and characteristic odor. It can be found naturally occurring in plant extracts or on its white crystalline synthetic form. It is found in *Lamiaceae* plant species, especially oreganos and thymes, which present antimicrobial, antioxidant and antiseptic properties [14–17]. It is considered safe in cosmetic formulations up to 0.5%, according to the Cosmetic Ingredients Review (CIR) and it is used as preservative in cosmetics and foods [18]. Moreover, TH antioxidant and antimicrobial properties allow cosmetic products to avoid the use of other chemical compounds as preservatives. The effects of TH are largely attributed to its antioxidant properties, via free radical scavenging thus enhancing endogenous antioxidant activities and chelation of metal ions [19]. The antioxidant activity provides an interesting therapeutic approach to restore skin homeostasis, maintaining its internal conditions relatively constant and stable, modulates the stratum corneum SC barrier function and prevents skin irritation [20].

Bacterial survival depends on membrane lipid homeostasis and the ability to adjust the lipid composition of bacterial cells in different environments. There are biochemical processes that underlie adjustments and are responsible for membrane phospholipid homeostasis in bacteria, controlling the movement of substances across the cell membrane [21]. These processes depend on proteins embedded in a lipid matrix that closely approximates a phospholipid bilayer.

The hydrophobic nature of TH facilitates its interaction with the lipidic bacterial membrane via direct binding with biomolecules, such as proteins, providing strong antimicrobial effect by altering its morphology and leading to bacterial death [22, 23]. Disruption of bacterial membrane will lead to cell disfunction and apoptosis, resulting in loss of intracellular contents. Some authors suggested that TH antibacterial action is due to the increased permeability of bacterial cell membranes [24]. However, other studies suggest that TH is responsible for the inactivation of enzymes implicated in synthesis of structural constituents [14]. The extent of bacteria membrane damage induced by a compound could be related to its intrinsic hydrophobicity. In the other hand, the slight hydrophilicity could enhance diffusion through the extracellular polysaccharide matrix and cause destabilization of bacterial biofilms [16].

With the aim to increase and prolong skin penetration into the hair follicle, without affecting the entire microbiota, PLGA NP containing TH (TH-NP) have been developed and optimized using the design of experiments (DoE) approach. Physicochemical characterization and biopharmaceutical behavior of optimized TH-NPs have been determined. Cytotoxicity, cellular uptake and antioxidant activity of TH-NP have also been assessed in human keratinocytes cell-line (HaCaT). In addition, antimicrobial therapeutic efficacy of this colloidal system was evaluated in vitro and ex vivo.

## Results

### Formulation characterization and optimization

The optimization of TH-NP was obtained by developing a full composite factorial design of five levels and three factors. Studied independent variables were the amount of TW and TH as well as pH formulation. The latter was chosen due both to Thymol pKa (pKa 10.6) which, as previously reported in other studies, can influence in the EE [25, 26].

The results of the DoE physicochemical characterization and the entrapment efficiency of TH-NPs are shown in Table 1. The average particle size (Z*av*) values ranged from 162 to 235 nm, being the polydispersity index (PI) comprised between 0.06 and 0.12. Based on the criteria for monodispersed systems (PI < 0.1), all the formulations presented homogeneity [27]. The surface charge of TH-NP, measured as Z-potential (ZP), ranged from − 22 to − 31 mV. This negative ZP is associated with negative surface charge associated with PLGA, the main NPs compound [25, 28–30]. Moreover, ZP is related to the stability of colloidal dispersions, for this reason, the developed formulations with values closest to − 30 mV, were considered the most stable. The encapsulation efficiency (EE) of designed formulations ranged from 71 to 83%.

**Table 1:** Values of the 2^3^ + star central composite factorial design, parameters, and responses

	Independent variables			Dependent variables/responses			
	TH	TW	pH	Z_av_ (nm)	PI	ZP (mV)	EE (%)
Factorial points							
F1	− 1	− 1	− 1	217.9 ± 1.2	0.112 ± 0.001	− 30.7 ± 0.9	76.35 ± 1.53
F2	1	− 1	− 1	234.9 ± 2.1	0.093 ± 0.004	− 29.9 ± 0.4	79.16 ± 0.18
F3	− 1	1	− 1	176.2 ± 1.0	0.075 ± 0.023	− 27.7 ± 1.3	76.67 ± 2.17
*F4*	*1*	*1*	− *1*	*174.0 ± 0.6*	*0.081 ± 0.012*	− *28.3 ± 0.8*	*80.07 ± 3.65*
F5	− 1	− 1	1	162.0 ± 0.4	0.072 ± 0.011	− 26.2 ± 0.3	79.64 ± 3.79
F6	1	− 1	1	163.5 ± 1.4	0.071 ± 0.009	− 23.6 ± 0.5	73.52 ± 1.83
F7	− 1	1	1	183.9 ± 0.4	0.087 ± 0.020	− 29.5 ± 0.1	78.41 ± 2.51
F8	1	1	1	172.4 ± 1.1	0.094 ± 0.009	− 26.6 ± 0.3	76.60 ± 5.60
Axial points							
F9	*1.68*	*0*	0	174.2 ± 0.6	0.061 ± 0.018	− 25.2 ± 0.4	77.01 ± 3.07
F10	− *1.68*	*0*	0	176.7 ± 1.1	0.083 ± 0.033	− 23.6 ± 0.3	73.55 ± 2.93
F11	*0*	*1.68*	0	187.1 ± 0.8	0.024 ± 0.006	− 23.1 ± 0.6	71.38 ± 0.62
F12	*0*	− *1.68*	0	167.4 ± 2.1	0.046 ± 0.021	− 26.1 ± 0.4	76.92 ± 2.47
F13	*0*	*0*	1.68	164.6 ± 1.1	0.057 ± 0.010	− 23.1 ± 0.8	72.44 ± 1.66
F14	*0*	*0*	− 1.68	202.6 ± 3.5	0.063 ± 0.045	− 22.5 ± 1.1	82.89 ± 6.01
Central points							
F15	0	0	0	175.4 ± 2.1	0.053 ± 0.013	− 24.5 ± 0.6	74.94 ± 1.77
F16	0	0	0	176.3 ± 1.9	0.072 ± 0.016	− 25.3 ± 0.7	78.14 ± 0.49

**Fig. 1:**
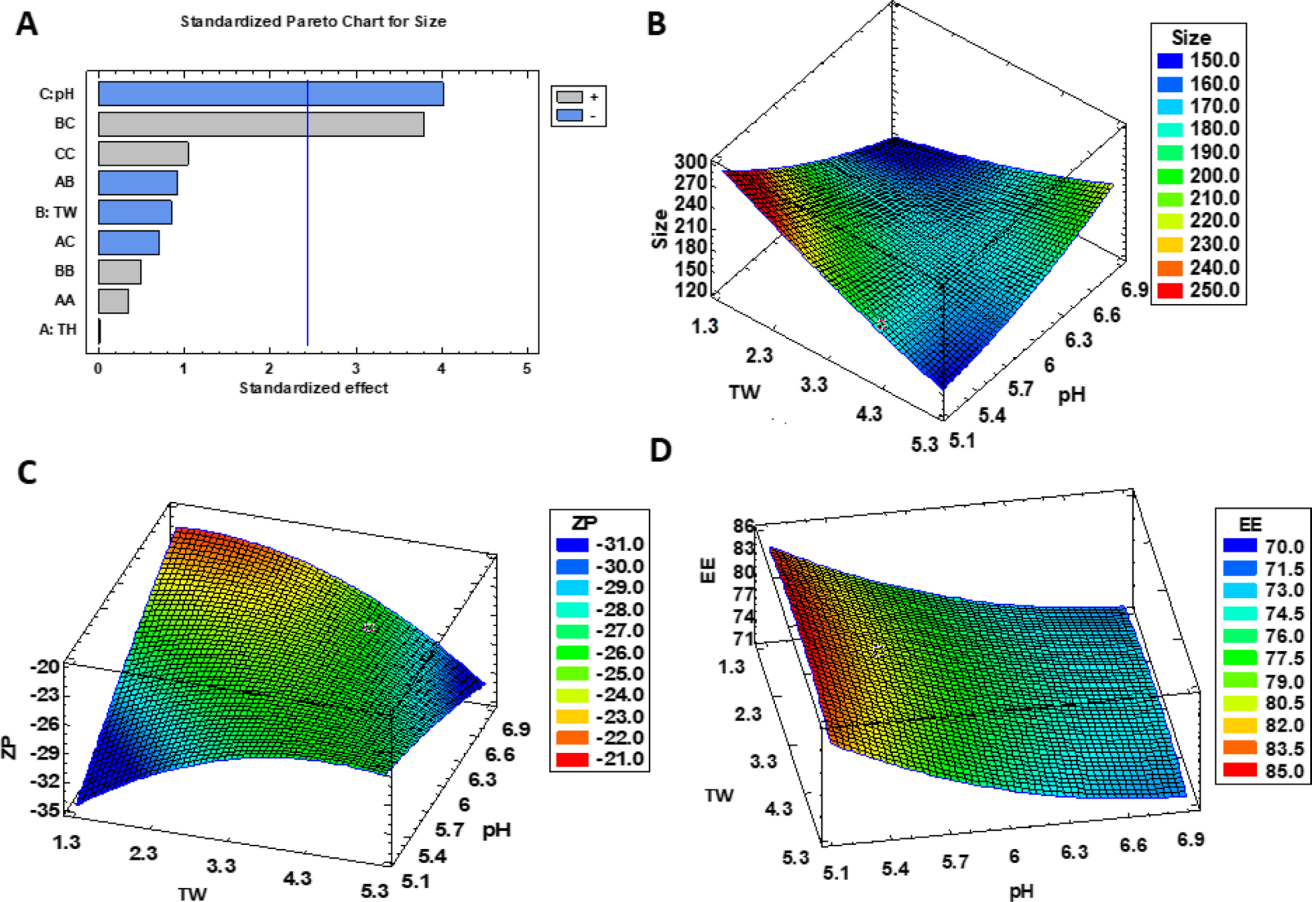
Factorial design with TH-NP fixed at 2.5 mg/mL TH: **A** Pareto’s chart for particle size (ANOVA) and surface response for **B** Z*av* particle size (nm), **C** ZP (mV) and **D** EE (%)

Surface response charts of the DoE, performed by Statgraphics® software, are shown in Fig. 1. The statistical analysis (ANOVA) only presented significant differences for the particle size (p < 0.01), influenced by both, pH of the aqueous phase and ratio of surfactant/pH (Fig. 1A). The responses at a fixed TH concentration (2.5 mg/mL) are illustrated for Z*av*, ZP and EE (Fig. 1B–D), respectively. Results show that the highest EE was achieved at the lowest pH, while for ZP, the absolute high values were reached when the pH and surfactant were simultaneously low or high. Considering all the evaluated parameters, formulation F4, containing 0.25% of TH and 0.4% tween 20 (TW) has been optimized to carry out further experiments.

### Morphology and stability of TH-NP

**Fig. 2:**
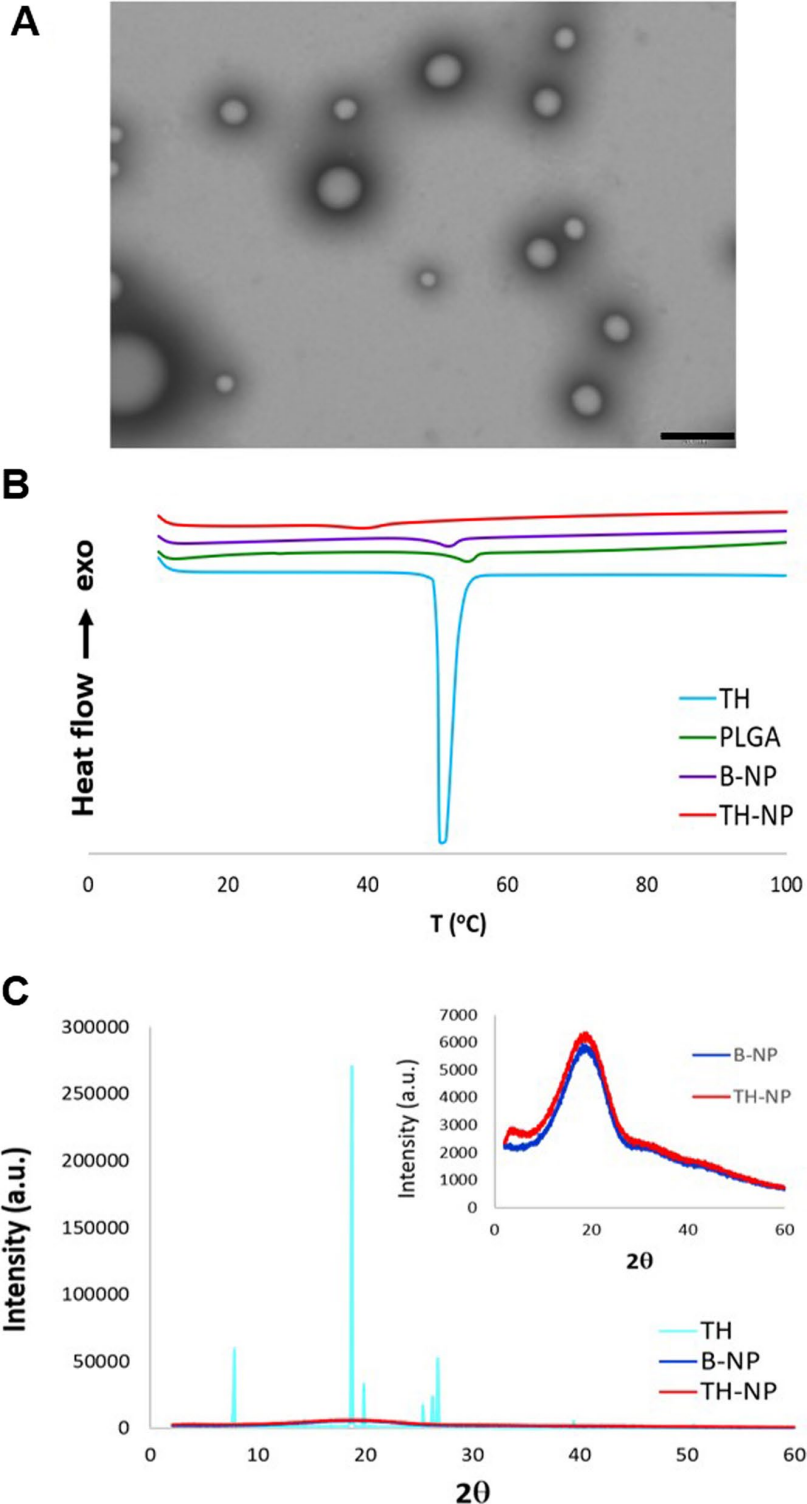
**A** Transmission electron microscopy image of TH-NP. Scale bar: 200 nm, **B** DSC thermograms of TH-NP, B-NP, and compounds separately, **C** X-ray diffraction patterns of TH-NP, B-NP, and compounds separately

The morphology of TH-NP was evaluated by transmission electron microscopy (TEM) and it is shown in Fig. 2A. Moreover, TH-NP maintained their structure for 1 month at 4 and 25 °C and additionally, for 12 months at 4 °C (Fig. 1 of SM). A small particle aggregation takes place after 12 months, which was confirmed by a slightly increased particle size, as indicated in Table 2.

**Table 2:** Physicochemical stability of TH-NP stored at different temperatures (4, 25 and 37 °C) measured at 0, 1, 3, 6 and 12 months

	t (months)	Z_av_ (nm)	PI	ZP (mV)	pH
	0	158.8 ± 1.7	0.068 ± 0.027	− 24.7 ± 1.0	4.20
4 °C	1	158.6 ± 1.8	0.108 ± 0.046	− 20.1 ± 0.6	4.07
	3	159.0 ± 2.1	0.101 ± 0.034	− 17.1 ± 0.1	3.90
	6	168.6 ± 1.7	0.152 ± 0.033	− 15.3 ± 0.1	3.68
	12	204.5 ± 1.3	0.231 ± 0.015	− 11.1 ± 0.7	3.61
25 °C	1	162.3 ± 0.4	0.119 ± 0.015	− 19.1 ± 0.1	3.91
	3	182.3 ± 1.4	0.145 ± 0.020	− 10.3 ± 0.2	3.64
37 °C	1	177.4 ± 1.2	0.128 ± 0.012	− 15.3 ± 0.6	3.32
	3	216.4 ± 2.8	0.205 ± 0.001	− 8.56 ± 0.3	2.95

This slight aggregation is also related with the decrease of ZP, since electrostatic forces between surface-charged NPs decrease when stored in aqueous media. Temperature showed to accelerate particle destabilization by decreased ZP. A slight decrease of the pH value was also observed, probably due to a partial hydrolysis of the polymer.

All these phenomena are in accordance with the predicted backscattering profile shown in Fig. 2 of SM, where TH-NP sedimentation was observed by the first left peak at the bottom of the vial, being reversible by agitation. Moreover, it can be observed that at 37 °C the signal greatly decreases, presenting TH-NP destabilization at higher temperatures. Additionally, EE was maintained at 4 and 25 °C for 6 months, whereas at 37 °C, decreased by 2.5-fold from the initial value. The parameters of all storage conditions were within stable criteria, presenting better short-term stability when stored at 4 °C. Moreover, samples presented no microbial growth within storage, confirming the preservative effect of TH.

### Interaction studies

The interactions between TH and PLGA, carried out by differential scanning calorimetry (DSC) and X-ray diffraction (XRD), are shown in Fig. 2. DSC thermograms of TH showed an endothermic peak at 52 °C, which corresponds to its melting transition (Fig. 2B). A minimal displacement of endotherm was presented by polymer alone and blank NPs (B-NP). However, TH-NP presented an onset peak displaced at 40 °C, due to TH-PLGA interaction. The XRD diffractograms (Fig. 2C) show TH on its crystalline form, expressed by the sharp diffraction peaks. TH-NP could be observed as non-sharp peaks, confirming that TH was dispersed in the polymer matrix in its amorphous form (molecular dispersion) and also showing a similar profile to B-NP.

### In vitro drug release

**Fig. 3:**
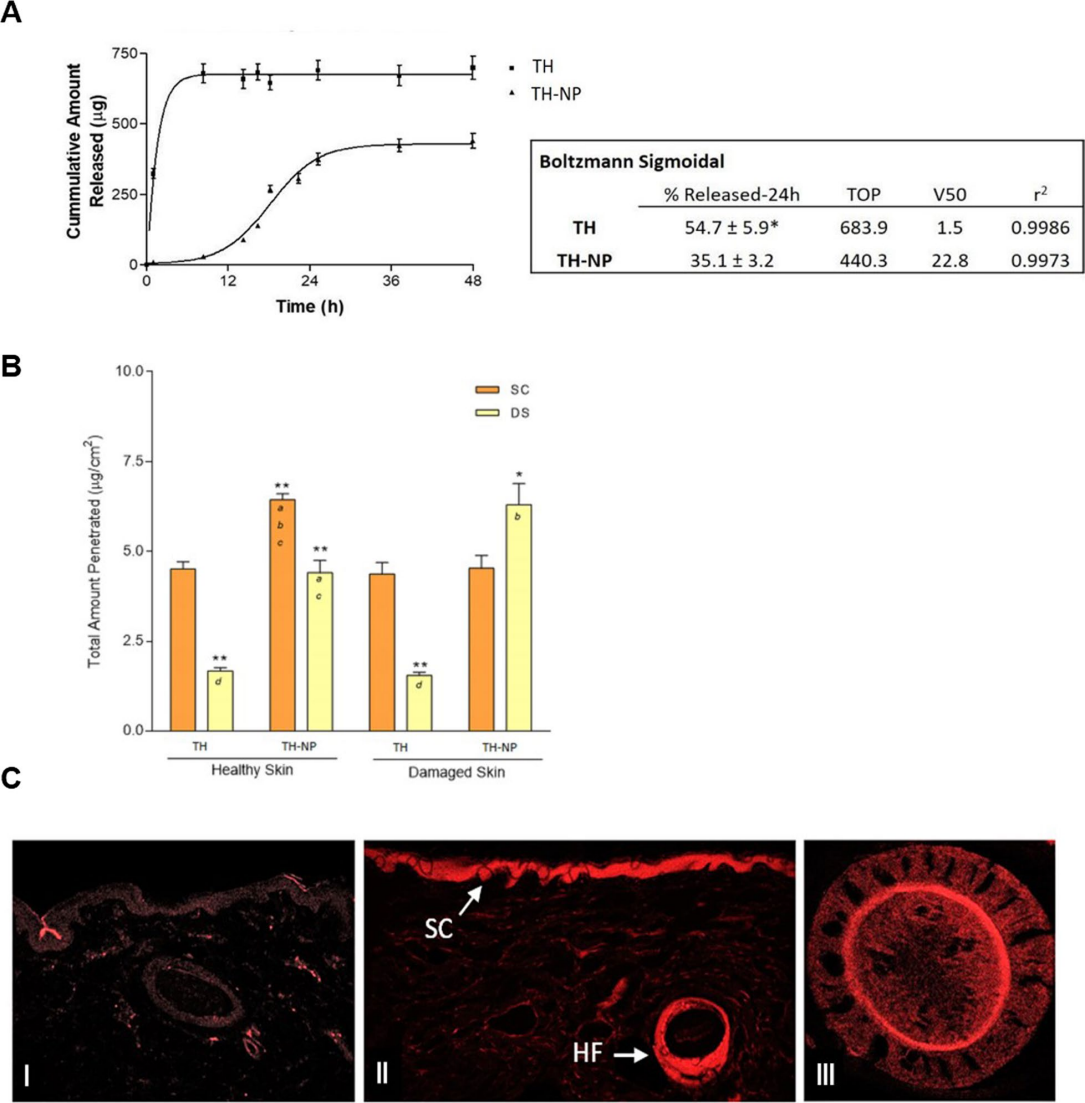
**A** Release profile of TH and TH-NP adjusted to Boltzmann Sigmoidal equation. Total amount released in 24 h expressed as % Mean ± SD (n = 3). Statistical analysis one-way ANOVA t-student test *p < 0.01, **B** Total amount of TH and TH-NP penetrated in 24 h in healthy and damage skin. SC: stratum corneum (tape stripping), DS: deep skin (extraction). Values represent the Mean ± SD (n = 3). Statistical analysis for each localization via one-way ANOVA Tukey's Multiple Comparison Test. Different letters inside the bars indicates significant differences between groups (*p < 0.01 and **p < 0.001), **C** Confocal microscopy images of pig skin R-TH-NP penetration after 24 h: **I** untreated skin control, **II** SC and hair follicle, **III** hair follicle cross-section

The in vitro release profile of TH from TH-NP against free TH, carried out in Franz diffusion cells, is shown in Fig. 3A. As can be observed, the release of free TH through the dialysis membrane was faster, while TH-NP provided a slow-rate prolonged release. Kinetic data was adjusted to Boltzmann Sigmoidal equation showing that TH reached 50% (V50) of total amount released within a short period of time (1.5 h), while TH-NP only achieved the same within 23 h. The total amount of TH released in 24 h was 55% and 35% for TH and TH-NP, respectively, presenting statistically significant differences (p < 0.01).

### Ex vivo skin permeation

The ex vivo skin permeation of TH and TH-NP were performed in healthy skin and, additionally, in damaged skin, where the SC was previously scratched to mimic skin barrier disorders. The corresponding kinetics of both skin types are shown in Table 3. The permeation flux (*J*) of TH and TH-NP were increased by 2.1- and 2.6-fold, respectively, on damaged compared to healthy skin, where all samples presented significant statistical differences (p < 0.001) between them. In both cases, damaged and healthy skins, TH presented significantly (p < 0.001) faster penetration rate compared to TH-NP, increased by 1.6 and 1.3-fold, respectively. The total amount penetrated (*Ap*) was significantly higher (p < 0.001) in damaged skin. In the other hand, the total amount retained inside the skin (*As*) was similar for both samples comparing healthy to damaged skins and there were significant differences (p < 0.001) comparing TH-NP with TH.

**Table 3:** Ex vivo skin permeation parameters

	Healthy skin		Damaged skin	
	TH	TH-NP	TH	TH-NP
J (μg/cm^2^/h)	12.68 ± 1.73	8.03 ± 0.55 *a*	26.43 ± 2.13 *a b d*	21.03 ± 0.92 *a b*
Kp (cm/h)	5.07E−03 ± 6.91E−03	3.21E−03 ± 2.19E−04 *a*	1.06E−02 ± 3.51E−04 *a b d*	8.41E−03 ± 3.69E−04 *a b*
Ap (μg/cm^2^)	106.43 ± 9.96	96.39 ± 12.68	272.36 ± 14.02 *a b d*	202.58 ± 11.65 *a b*
As (μg/cm^2^*)*	6.19 ± 1.45	10.85 ± 1.12 *a c*	5.92 ± 0.92	10.83 ± 2.13 *a c*
*At (μg/cm*^***2***^***)***	*112.61 ± 11.41*	*107.24 ± 13.80*	*278.28 ± 14.93 a b d*	*213.78 ± 13.78 a b*
SSD (p < 0.01)	*a*	*b*	*c*	*d*

J: flux, Kp: permeability constant, Ap: total amount penetrated, As: total amount retained inside the skin, At: total amount penetrated and retained inside the skin

The total amount of thymol penetrated within 24 h (Fig. 3B), was split into stratum corneum (SC) and deep skin (DS). The amount found in the SC was the same for TH on both skin types and similarly to TH-NP on damaged skin. However, for TH-NP, the retained amount was significantly higher (p < 0.01) on normal skin, in agreement with the slow-rate penetration, presenting delayed entry of the particles. In the other hand, it can be observed that TH-NP presented significantly (p < 0.001) higher amounts retained in DS on both skin types, compared to TH. Meanwhile, TH-NP on damaged skin was significantly higher than normal skin (p < 0.01).

Ex vivo skin permeation route was studied by confocal microscopy using rhodamine-labelled TH-NP (R-TH-NP) after 24 h of permeation (Fig. 3C). The results obtained showed that R-TH-NP successfully penetrated inside the skin hair follicle, where acne pathogen infection and inflammation occur. The image (Fig. 3C_II) illustrates that R-TH-NP were found concentrated in the hair follicle and presented delayed entry accumulation in the SC.

### Cytotoxicity in HaCaT cells

**Fig. 4:**
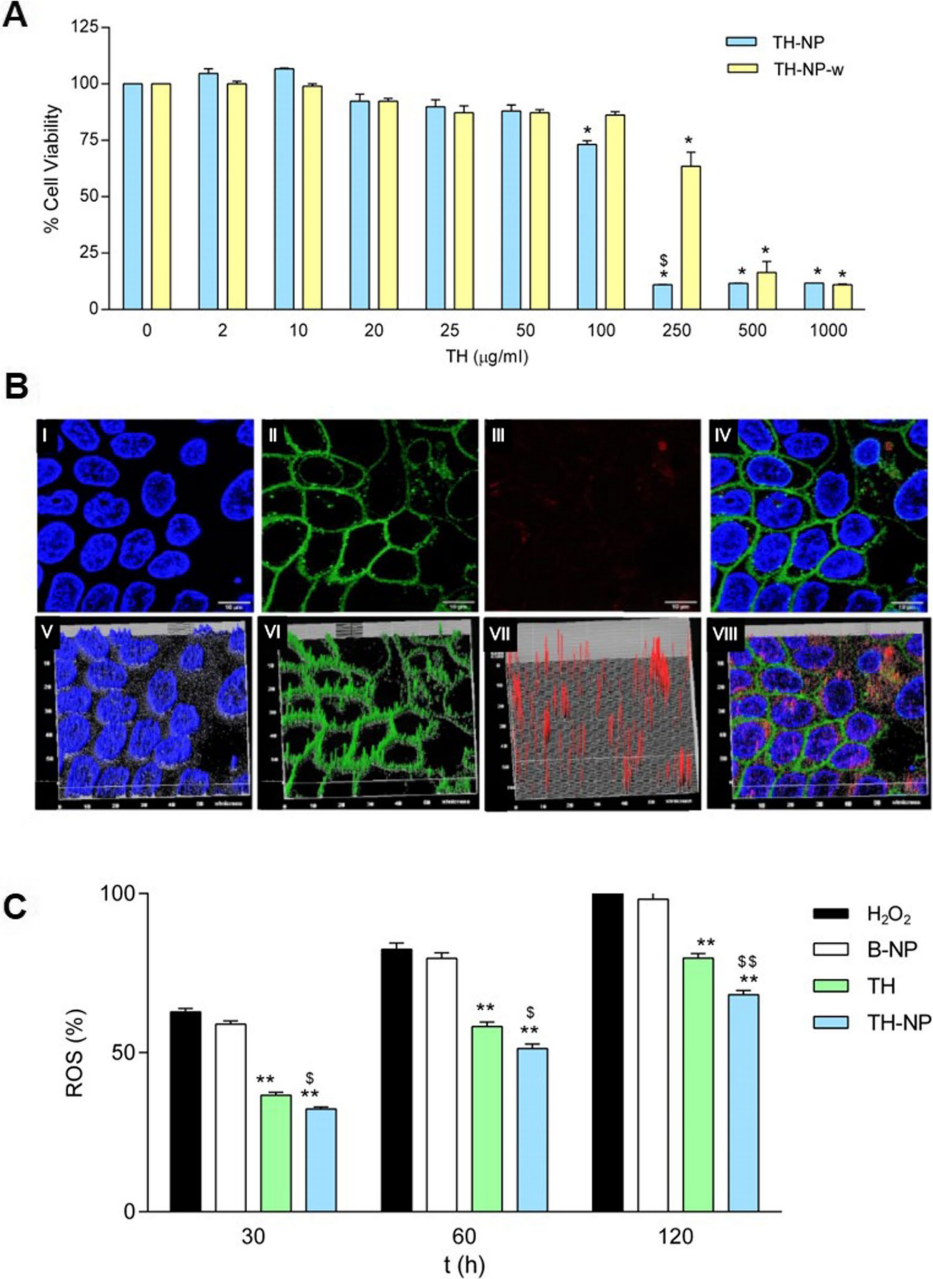
**A** Cell viability of HaCaT keratinocytes after incubation for 24 h with TH-NP and the washed NPs (TH-NP-w) at different concentrations. Cell viability was assayed by the MTT reduction method; 100% viability was set with the values obtained with the untreated control cells. Values represent the Mean ± SD (n = 3). Statistical analysis one-way ANOVA Tukey ‘s Multiple Comparison Test, *p < 0.01 versus control and ^$^p < 0.01 versus TH-NP-w, **B** Confocal microscopy analysis of HaCaT cells incubated with R-TH-NP: (**I**) nuclei; (**II**) membranes; (**III**) R-TH-NP; (**IV**) merged (I), (II), and (III), respectively; (**V**–**VIII**-h) 3D-plot of (**I**)-(**IV**), respectively, **C** Time course analysis of ROS production induced by H_2_O_2_ (2 mM) in HaCaT cells treated with TH or TH-NP. The ROS production (100%) was set with the values obtained with cells challenged with H_2_O_2_ for 2 h. Data were expressed (%) as the Mean ± SD (n = 8). Statistical analysis was performed by one-way ANOVA Tukey’s Multiple Comparison Test, **p < 0.001 versus controls at 2 h; ^$^p < 0.05 and ^$$^p < 0.001 between TH and TH-NP treated cells

The cytotoxicity of TH-NP was evaluated on HaCaT cells, incubated for 24 h with concentrations up to 1 mg/mL (Fig. 4A). Results showed that TH-NP was not cytotoxic at concentrations up to 50 µg/mL, as cell viability was kept close to the untreated control cells. A 20% reduction in cell viability was observed at 100 µg/mL and close to 90% reduction at concentrations ≥ 250 µg/mL. Different results were obtained with washed nanoparticles (TH-NP-w), which were not cytotoxic at 100 µg/mL and caused only a 25% reduction in cell viability at 250 µg/mL. Differences between TH-NP and TH-NP-w indicate that the presence of free TW in the samples could cause toxicity to HaCaT cells.

### Cellular uptake of TH-NP

The cellular uptake of R-TH-NP (20 µg/mL) was analyzed in HaCaT cells. At this nanoparticle concentration, cell viability was over 90%. After 2 h incubation, fluorescence was detected by confocal microscopy in cells treated with R-TH-NP but not in untreated control cells (Fig. 4B). The cell membrane and the nucleus are represented as green and blue, respectively. In the merged images, it can be observed that the internalized nanoparticles were mainly localized in the cytoplasm.

### In vitro antioxidant efficacy in HaCaT cells

The antioxidant activity of TH and TH-NP, performed in HaCaT cells, was successfully achieved by reducing the amount of reactive oxygen species (ROS) generated. While B-NP did not present activity, TH and TH-NP showed a 20% and 32% of ROS reduction, respectively, within 2 h treated with H_2_O_2_ (Fig. 4C). Moreover, TH-NP was statistically significant compared to TH.

### In vitro antimicrobial efficacy

The minimal inhibitory concentration (MIC) was determined for TH and TH-NP on *S. epidermidis* being both 512 µg/mL. For *C. acnes,* TH and TH-NP displayed the same MIC and minimal bactericidal concentration (MBC) values, being 250 µg/mL and 400 µg/mL, respectively. Therefore, no differences were observed in the concentrations between samples. However, there were relevant differences between different bacterial strains. In the other hand, clindamycin, a strong antibiotic commonly used to treat severe acne, presented MIC < 2 µg/mL for both microorganisms. For this reason, clindamycin is able to treat acne. However, it also affects the healthy resident bacteria of the skin.

**Fig. 5:**
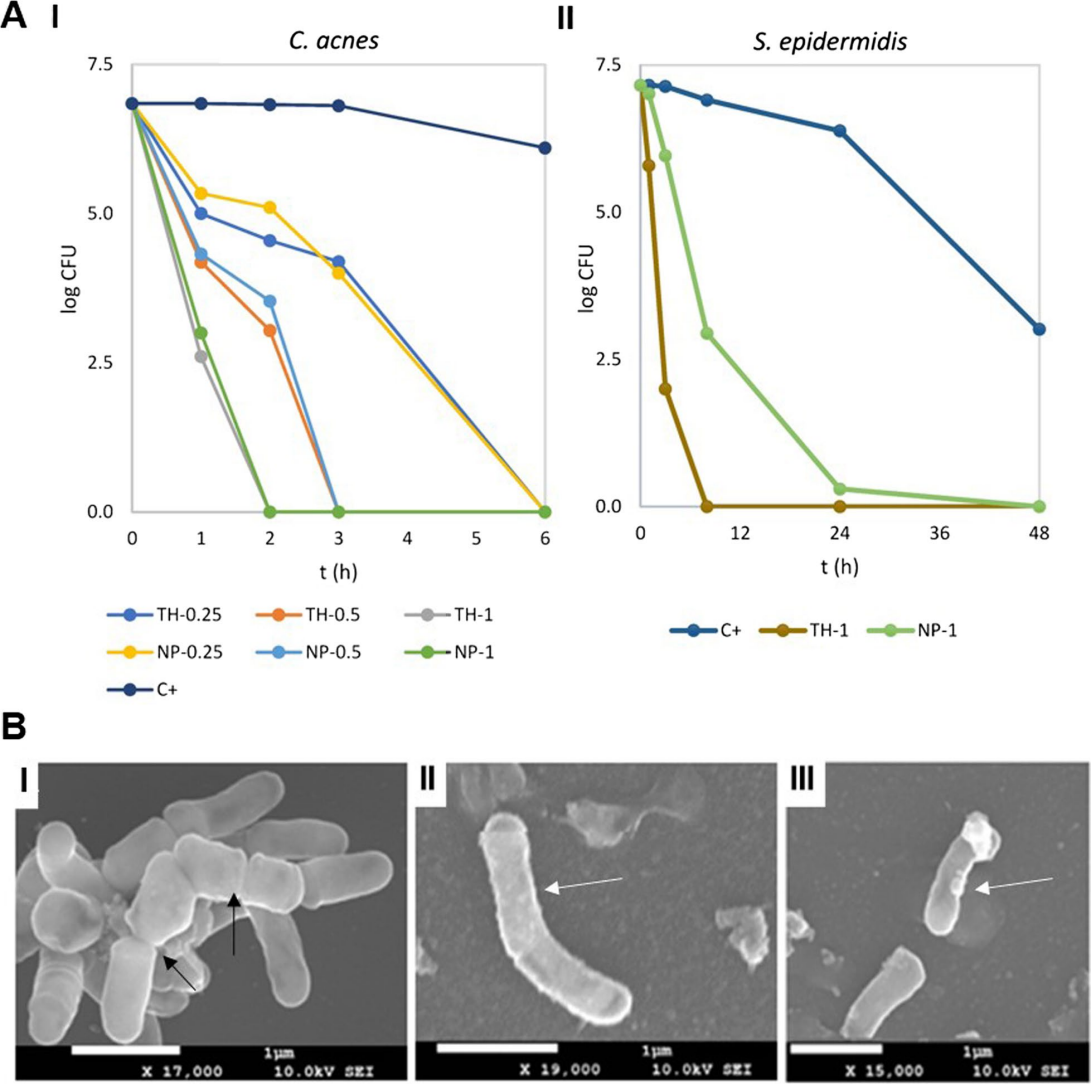
**A** Bacterial viability reduction of (**I**) *C. acnes* 6 h contact with TH or TH-NP (dose dependent) at 0.25, 0.5 and 1 mg/mL, (**II**) *S. epidermidis* 48 h contact with THF or TH-NP at 1 mg/mL. Data is expressed as log_10_CFU of mean values, **B** SEM micrographs of *C. acnes* (**I**) control and treated for 1 h with (**II**) TH or (**III**) TH-NP. Black arrows indicate bacteria division and white arrows membrane disruption

The bacterial viability reduction, evaluated by determination of decimal reduction time (D), treated with TH or TH-NP in a timely manner, is illustrated in Fig. 5A. In the case of *C. acnes,* the decrease of viable bacteria correlated with the applied dose. Although the effect of TH and TH-NP were similar, the activity of TH-NP was slightly sustained at lower dosages. At the MIC concentrations, they present minimal reduction activity, whereas, at concentrations higher than MBC, the reduction was boosted for all tested samples. Meanwhile, *S. epidermidis* presented very slow viability reduction when incubated with TH and TH-NP at twice the MIC (Fig. 5B), the same highest concentration tested for *C. acnes*. It can be observed that TH completely reduced *S. epidermidis* viability within 8 h, whereas TH-NP treated cultures still presented living colonies within 24 h.

Data of decimal reduction time (D), the time taken to reduce a decimal part of the bacterial viability, is shown in Table 4. In the case of *C. acnes,* differences could be observed comparing the variable dosages applied. However, variations between TH and TH-NP were not detected. In the case of *S. epidermidis*, TH-NP presented statistically significant differences compared to TH. Moreover, comparing the activity between both microorganisms, at the same dosage, only TH-NP presented statistically significant differences for *S. epidermidis* against *C. acnes*.

**Table 4:** Decimal reduction time (D) for *C. acnes* and *S. epidermidis* viability

		TH (mg/mL)					TH-NP (mg/mL)					
		0.25		0.50	1.00		0.25		0.50		1.00	
*C. acnes*												
*r*^*2*^		0.9654		0.9743	0.9812		0.9964		0.9984		0.9949	
*k (logCFU/h)*		1.00 ± 0.11		2.03 ± 0.19	3.48 ± 0.08		1.02 ± 0.12		1.96 ± 0.43		3.44 ± 0.03	
*D (min)*		**60.5 ± 6.5**		**29.6 ± 2.7**	**17.3 ± 0.4**		**59.0 ± 7.0**		**31.4 ± 6.9**		**17.4 ± 0.1**	
***S. epidermidis***												
*r*^*2*^					0.9944						0.9886	
*k (logCFU/h)*					1.75 ± 0.02						0.54 ± 0.01	
***D (min)***					**34.2 ± 0.4**						111.1 ± 2.9*^$^	

CFU: colony forming units. One-way ANOVA (t test): statistically significant differences *p > 0.001 versus TH, ^$^*p* > *0.0001 against C. acnes, same dosages*

Moreover, the structure of *C. acnes* (Fig. 5B), evaluated by scanning electron microscopy (SEM), presents a rod-shape and smooth membrane. Treatment with TH or TH-NP resulted on elongated cells, thickened cell envelope, and blebs formed on the surface.

### Ex vivo antimicrobial efficacy

**Fig. 6:**
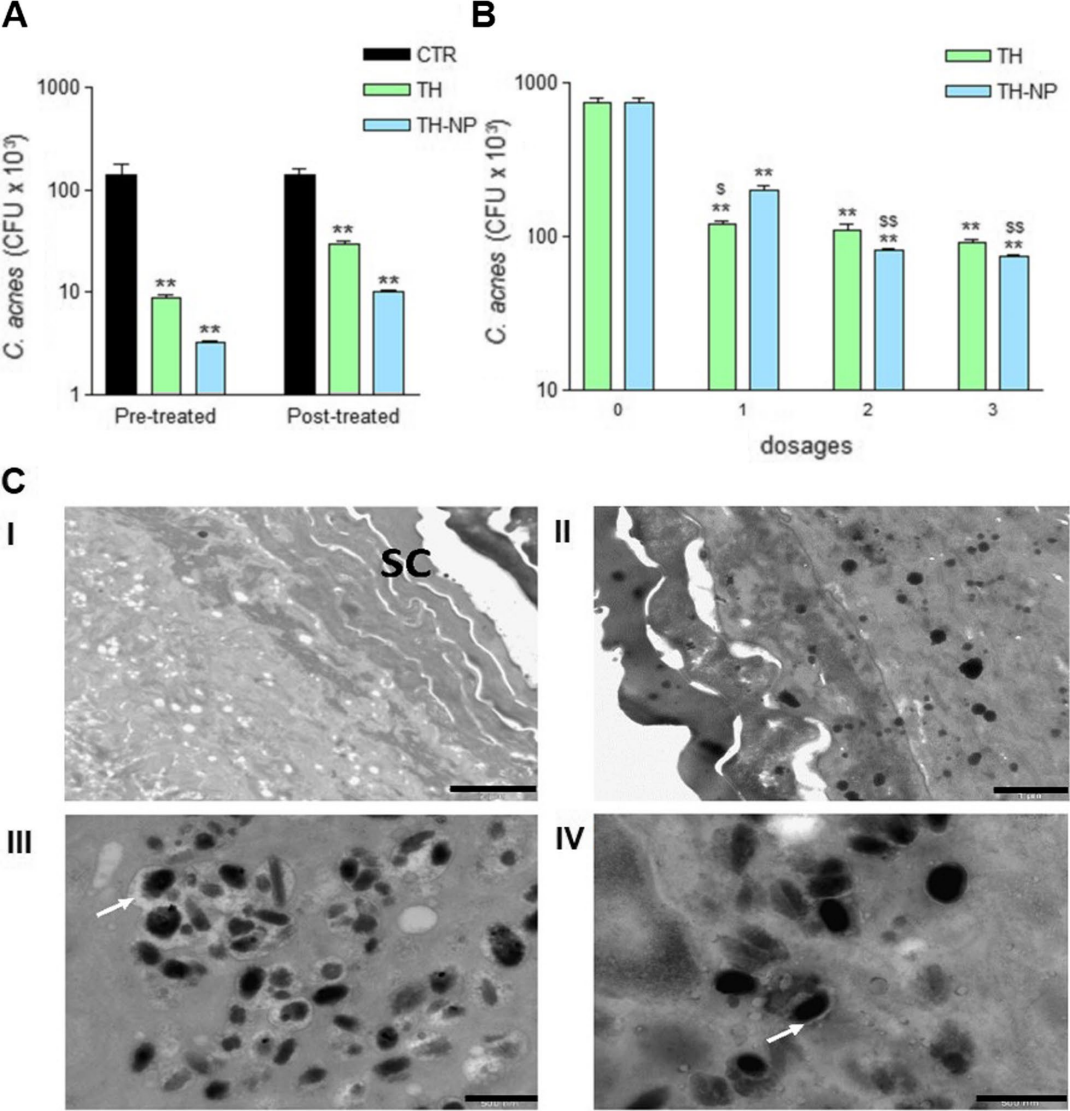
**A** Bacterial viability on ex vivo treated skin with TH or TH-NP prevention, **B** Bacterial viability on ex vivo treated skin with TH or TH-NP dose-dependent treatment with 3 applied doses at times 0, 12 and 18 h of incubation. Values represent viable count of *C. acnes* as the Mean ± SD (n = 3), **C** TEM images of normal human ex vivo skin: (**I**) untreated, (**II**) inoculated with *C. acnes (black)*, treated with (**III**) TH and (**IV**) TH-NP. White arrows indicate the loss of bacterial intracellular material. Scale bars: 2 µm (**I**), 1 µm (**II**) and 500 nm (**III** and **IV**). Statistical analysis one-way ANOVA Tukey’s Multiple Comparison Test represent **p < 0.001 compared to control and ^$^p < 0.01/^$$^p < 0.001 comparing TH and TH-NP.

The ex vivo antimicrobial efficacy of TH and TH-NP on *C. acnes* skin inoculated, as prevention or treatment for 24 h, were successfully determined. In both studies, all samples presented significant differences against the control (**p < 0.001). The activity was found greater as prevention than treatment (Fig. 6A), where TH-NP presented higher activity than TH, but no statistically significant differences were observed. In the other hand, for the dose-dependent treatment (Fig. 6B), administration of a single dose showed significant differences between TH and TH-NP (^#^p < 0.05).

Although TH-NP had lower effectiveness within one dose, they performed greater activity than TH when 2 or 3 doses were applied (^$$^p < 0.001). Meanwhile, TH did not show statistical differences comparing the 3 dosage protocols. Moreover, the highest efficiency of the experiment was achieved by 3 doses of TH-NP.

The simulation of skin infection and treatment was carried out on ex vivo fresh human skin explants, by inoculating *C. acnes* for 16 h, followed by 8 h treatment with TH or TH-NP. Morphology of *C. acnes* can be observed in Additional file 1: Figure S3. Moreover, Figure 6C illustrates the untreated skin and the *C. acnes* inoculated and penetrated within the skin layers. The treatment with TH demonstrated a fast and strong activity towards the bacteria membrane, presenting surrounding it, a great loss of intercellular material which may indicate damaged membrane. In the case of TH-NP, a slower effect with less amount of cellular leakage could be observed. The minor effects of TH-NP within 8 h of treatment might be related to the slow-rate release and penetration.

## Discussion

In the present study, TH was successfully loaded into PLGA NP. Developed TH-NP presented suitable physicochemical parameters with excellent stability and high EE. DoE analysis showed that Pareto’s diagram and surface responses indicated that only the pH of the aqueous phase and the ratio of pH/TW induced statistically significant differences on Z_av_, being the effect of these variables not significant on the EE. The surfactant usually shows statistically significant influence on the EE and morphometry of polymeric NP, depending on the amount applied [27]. TW has good permeability profile and its amphiphilic properties may control the interactions between the active compound and the biopolymer carrier [31]. Interaction studies showed that encapsulated TH was present on its amorphous form and the thermal transition was affected by TH-polymer interactions [25]. Analyzing the stability of TH-NP for the first 3 months, an increase of particle size and a decrease in ZP, depending on the storage temperature, were observed. The decrease of ZP induced TH-NP instability in aqueous medium, due to a decrease of electrostatic forces between surface-charged NP, generating in some cases, a slight particle aggregation. Moreover, TH-NP at high temperature, presented sedimentation that was reversed by agitation, as also observed by other authors [32]. TH-NP stored at 4 °C have shown outstanding short-term stability in aqueous solution. However, in order to extend TH-NP stability, either incorporation in a semi-solid formulation or freeze-drying are recommended [27, 33]. In addition, it has been shown that TH-NP did not present microbial contamination along storage, confirming TH antimicrobial preservative activity.

Biopharmaceutical behavior of TH-NP presented a sustained in vitro release profile, while TH reached the steady state very fast. The ex vivo skin rate of TH was higher than TH-NP by 1.6 and 1.3-fold, respectively, being faster on damaged than healthy skin. As previously described [34], these results confirm that the external hydrophobic skin surface was altered, thus enabling substances to penetrate faster and easier through this barrier. The enhanced penetration of TH and TH-NP through the skin may be due to TH properties as well as the TW properties. On the one hand, TH is a terpene and these kind of compounds are known to be skin penetration enhancers, by impairing the lipids of the SC [35]. On the other hand, TW, an anionic surfactant, also provide enhanced permeability through phospholipid membranes, inducing some damage to epidermal membranes which decrease skin resistance towards the diffusion of the active. Some authors also explained that the mechanisms of TW could be attributed to an improvement of the thermodynamic activity adsorption and fusion due to micellar complexation, or decreasing the SC hindrance or modification of its intracellular lipid barriers [36]. The amount of TH retained inside the skin was higher for TH-NP in both (healthy and damaged) skin types. The only difference between healthy and damaged skins was the highest amount in the SC and DS, respectively. The lesser amount of free TH against TH-NP retained inside the skin was probably due to its fast-rate penetration. In agreement with TH-NP slow-rate penetration, confocal microscopy study confirmed that TH-NP presented delayed entry, accumulating into the layers of the SC. This means that when the SC was disrupted, the flux of NPs penetration improved, and therefore, the amount inside the deeper layers of the epidermis and dermis increased. Interestingly, TH-NP skin penetration route was through the hair follicle, exactly where acne occurs. According to previous authors [37], the physicochemical properties of the active compound as well as the barrier properties of the hair follicles define the penetration route (intrafollicular or transfollicular). Polymeric NPs preferentially accumulate in the follicular entry, in a time dependent manner, where the skin penetration through the hair follicle is size dependent [8]. In this way, particles with average diameter of 200 nm are likely to penetrate faster than micro-sized particles or free molecules. Therefore, the smaller the particle size, the higher would be the accumulation into the hair follicle, and thus achieving lower permeability rates [8, 37]. Studies carried out by Yukuyama et al. [38], indicated that NP stored in the hair follicle will be cleared by hair growth or sebum production. Zhu et al.[39], developed PLGA TH microparticles as antimicrobial agents for food preservative application, containing particle size ranging for 20 to 70 µm. Due to the fact of the particle size selectivity for follicle entry, our developed TH-NP would be more efficient for penetrating the hair follicle to treat acne. Furthermore, other authors stated that the reservoir of the hair follicle could store actives 10 times longer than the reservoir of the SC, and also, that hair follicle under movement (in vivo) would improve NP penetration [37, 38]. Recent studies confirmed that diffusion of polymeric NPs only crossed the SC reaching the viable epidermis only after needle puncture [37]. This could be also observed, by confocal microscopy in healthy skin, where TH-NP were not found beyond the SC, unless inside the hair follicle. For all these reasons, TH-NP are more efficient for acne treatment due to the prolonged penetration and release inside the hair follicle.

In the HaCaT cell line, TH-NP at low concentrations did not alter cell viability, presenting no cytotoxicity. The cellular uptake images showed most of the NPs in the cytosol but also some particles reached the nucleus within only 2 h. This would enable TH-NP to perform its antioxidant activity inside the cells to improve the skin healing process on acne lesions [20]. This activity was confirmed by reducing the ROS generated, since TH-NP improved significantly compared to TH. Moreover, prolonged release of TH as well as increased stability of TH-NP may also favor enhanced antioxidant activity.

The antimicrobial activity of TH and TH-NP against *C. acnes* was similar and successfully demonstrated in vitro and ex vivo. The in vitro activity increased with high concentrations of TH-NP, and they presented the decimal reduction of bacterial viability within 60, 30 and 17 min, for MIC, 2 × MIC and 4 × MIC, respectively, confirming dose-dependent activity. Additionally, the concentrations used above MBC completely reduced all CFU very fast. For the ex vivo experiments, simulating an acne infection inside the skin explant, samples resulted more efficient for prevention than treatment, despite, no significant differences between samples could be observed. This can be explained by the fact that part of the amount of the formulations applied were retained on the SC, and therefore, products had a direct contact with *C. acnes* when this was inoculated onto the skin. Meanwhile TH penetrates faster throughout the skin, providing only an immediate effect. For TH-NP, the effectiveness was higher when multiple dosages were applied onto the skin providing a slightly sustained effect. These results are in accordance with those observed in the biopharmaceutical studies. The same behavior was observed by electron microscopy in the ex vivo images, where the effect of TH on the bacterial membrane was found stronger than TH-NP, for 8 h contact inside the skin. For this reason, it could predictably act in the same way on skin microbiota and therefore, TH-NP would constitute a less aggressive and more efficient system for such treatment. The in vitro SEM images showed modified cells, with greater wall thickness and the development of blebs [40]. This observation agrees with the previously mentioned mechanism of antimicrobial activity, by triggering loss of intercellular nutrients.

Concerning *S. epidermidis,* a more resistant type of microorganism, it presented very slow viability reduction in contact with TH and TH-NP, compared to *C. acnes*, even tested at the highest concentration. Growth was abolished within 8 h for TH, while for TH-NP this effect was observed over 24 h. Therefore, if these NP were administered in vivo for acne skin treatment, at the same concentrations, it would be less effective towards the entire skin microbiota, being at the same time a strong bactericidal against *C. acnes.* This favors the desirable microbiota-friendly activity, where the antimicrobial ingredient will not alter the good functionality of the healthy skin, acting efficiently against *C. acnes* and only mediating *S. epidermidis* proliferation. Another proof of concept was previously stated by other authors [41] providing evidence that microbiota shifts notably during puberty, increasing predominance of *Cutibacterium* species and decreasing abundance of *Staphylococcus* species. Meanwhile, in adulthood it remains unstable due to constant external environmental changes, suggesting mutual beneficial microbiome-host interactions. As a complement, the MICs showed that the concentration needed as bactericidal against *C. acnes* was lower than the minimal concentration needed to inhibit *S. epidermidis* growth. This means that for in vivo conditions, the desirable effect for acne treatment would maintain the skin balanced. In the case of clindamycin, the MIC for *C. acnes* and *S. epidermidis* was confirmed to be strongly efficient at a very low dose. This powerful activity is the main challenge with this type of drug, since it completely abolishes all the microbial content of the skin, therefore, treating it by unbalancing the healthy conditions for the microbiota, thus, leading to microbial resistance. For this reason, in this study we managed to confirm an effective activity of a natural active, at higher concentration, but that can maintain the microbiota function to rebalance the skin conditions, maintaining it healthy upon the treatment.

From another point of view, since the route of penetration of polymeric TH-NP into the skin was through the hair follicle, the observed activity will be performed directly on the acne lesion. Therefore, TH-NP perform a protective effect on the healthy skin microbiota, along with extending the retention and release of TH, directly to the site of the infection with prolonged activity. Moreover, the sebum content on the hair follicles could facilitate absorption and release of TH from TH-NP by hydrophobic interactions. Nevertheless, since PLGA skin metabolism occurs by biodegradation into its monomers (lactic acid and glycolic acid), these compounds may help to modulate the skin pH, which would contribute to the sebum control.

## Conclusions

TH was successfully encapsulated into PLGA NPs with particle size below 200 nm and high EE with suitable stability. Moreover, TH-NP solution did not present microbial growth under a storage period of 12 months, due to antimicrobial properties of TH. Therefore, they can act as natural preservative system. TH-NP presented a sustained release and slow-rate penetration on skin, through the hair follicle, with higher amounts retained inside the skin, compared to TH free. Moreover, TH-NP showed outstanding antimicrobial activity in vitro and ex vivo against *C. acnes*, with minor effects towards *S. epidermidis*, which promises to be a great microbiota-friendly candidate for acne treatment. Additionally, TH-NP adhered into the SC layers would provide good protection on the acne lesions against external microbial aggressors. Moreover, the cellular uptake of TH-NP has also improved the antioxidant activity in keratinocyte cells, which would be promising on cell regeneration on the healing process of the acne lesion. Therefore, TH loaded nanostructured system has been successfully developed and physicochemically characterized demonstrating excellent properties for acne topical treatment.

## Methods

### Materials

PLGA Resomer® RG 504H (consisting of a carboxylic terminal group, molecular weight 38,000–54,000 Da and molar ratio lactide:glycolide 50:50) was purchased from Boehringer Ingelheim (Ingelheim, Germany). Thymol (TH), Tween 20 (TW) and acetone were purchased from Sigma-Aldrich (Spain). Milli-Q water was obtained from a double distilled Millipore system. All chemicals and reagents used were analytical grade (HPLC).

### Preparation of TH-NP

TH-NP containing a matrix structure (nanospheres) were obtained by solvent displacement evaporation, described by Fessi et al. [42]. Briefly, an aqueous phase containing TW and an organic phase were prepared. The organic phase was made by dissolving PLGA and TH in acetone, and it was added dropwise into the aqueous phase under continuous stirring. Finally, in order to evaporate the organic solvent, a rotatory evaporator (Buchi, Switzerland) was used at 30 °C under constant pressure, obtaining TH-NP dispersed in water [43–45].

### Optimization of TH-NP

TH-NP were optimized using the design of experiments approach (DoE). A full factorial central design of five levels and three factors was applied in order to reduce the number of experiments [46]. This experimental design consisted of 16 formulations with variable factorial points (− 1/ + 1), axial points (− 1.68/ + 1.68) and central points (0), each involving 8, 6 and 2 formulations, respectively. The concentration of active (TH), surfactant (TW) and the pH of the aqueous phase were selected as the independent variables (Table 5). PLGA was fixed to 9 mg/mL for the entire experiment. The effect of the independent on the dependent variables (morphology, z-potential and encapsulation efficiency) has been analyzed [47].

**Table 5:** Factors and levels of DoE independent variables

Factors	Levels				
	− 1.68	− 1	0	1	1.68
TH (mg/mL)	1.16	1.5	2.0	2.5	2.84
TW (mg/mL)	1.32	2.0	3.0	4.0	4.68
pH	4.32	5.5	6.0	6.5	7.68

### Physicochemical characterization of TH-NP

Z_*av*_ and PI were determined by photon correlation spectroscopy, using a ZetaSizer Nano ZS (Malvern Instruments; Malvern, UK). The surface charge, measured as ZP, was determined by electrophoretic mobility using the same instrument. The morphology of the particles was accessed by TEM (transmission electron microscopy, JEOL JEM1010, Tokyo, Japan), using Megaview III (Soft Imaging Systems, GmbH, Münster, Germany). The negative staining was carried out with 2% uranyl acetate.

Quantitative analysis was performed by reverse-phase high-performance liquid chromatography (HPLC) by a modification of the method described previously [48]. Studies were carried out in Acquity Waters System with UV detector, using a Kromasil® column (C18, 5 μm, 150 × 4.6 mm). The mobile phase consisted of acetonitrile:water under gradient conditions of 30:70/58:42/30:70 during 20 min. TH was determined at wavelength of 275 nm.

The encapsulation of TH was measured indirectly by quantification of unloaded amount. Samples were diluted 1:10 in Milli-Q water:ethanol (90:10) and centrifuged (Centrifuge 5415C, Geratebau Eppendorf GmbH, Engelsdorf, Germany) for 10 min at 14,000 rpm, using Millipore filter device (Amicon® Ultra, 0.5 mL 100 K, Merck Millipore Ltd., Carrigtwohill Co. Cork IRL). The filtered fractions were quantified by HPLC, and the EE was determined by Eq. (1):

1 $$\mathrm{EE}=\frac{Ci-Cs}{Ci}\cdot 100$$

where *Ci* is the initial concentration of the active and *Cs* is the concentration of the unloaded amount found in the filtered fraction.

### Interaction studies

Interaction studied were carried out by previous ultracentrifugation of the samples at 15,000 rpm during 30 min of TH-NPs (Beckmann-Coulter ultracentrifuge). The possible interactions between TH and PLGA were assessed by differential scanning calorimetry (DSC). Thermograms were obtained on a DSC823e System (Mettler-Toledo, Barcelona, Spain). A pan with indium (purity ≥ 99.95%; Fluka, Switzerland) was used to check the calibration of the calorimetric system and an empty pan was used as a reference [25]. Samples were heated from 10 °C to 100 °C at 5 °C/min under a nitrogen atmosphere. Data were evaluated from the peak areas with Mettler STARe V 9.01 DB software (Mettler-Toledo). The physical state (amorphous or crystalline) of TH and TH-NP was analyzed by X-ray diffraction (XRD). Samples were sandwiched between 3.6 µm films of polyester and exposed to Cu K α radiation (λ = 1.5418 Å) with work power (45 kV, 40 mA). Diffractograms were recorded on a PANalytical X’Pert PRO MPD θ/θ, powder diffractometer of 240 mm of radius, using PIXcel detector (active length = 3.347°). The measure time was defined 200 s per step, 2θ/θ scans from 2 to 60°2θ with a step size of 0.026°2θ [49].

### Stability of TH-NP

The physicochemical stability of the optimized formulation was followed during storage at different conditions: 25 and 37 °C for 3 months and 4 °C for 12 months. The stability was studied by measuring backscattering of near-infrared pulsed light (λ = 880 nm), bottom-to-top of the turbiscan cell containing TH-NP, using optical analyzer Turbiscan®Lab expert (Formulaction, L’Union, France), to predict the behavior of the NPs in solution. Additionally, measurements of Z_av_, PI, ZP and TEM images were also monitored at selected times. The EE stability was also measured at 6 months of storage.

To analyze the microbial preservative activity of TH during storage, samples stored for 6 months at room temperature and 12 months at 4 °C where used. For direct measurement, 0.1 mL of each sample was transferred into the plates or, additionally, samples where diluted 1:10 in neutralizing solution (Berens Cosmetic Diluent, Scharlab, UK), then 1 mL was transferred into the plates. The total viable count was carried out by inclusion on TSA (Tryptone Soy Agar, Oxoid, UK) for bacteria or Sabouraud Dextrose Agar (Oxoid, UK) for fungi and yeasts. Plates were incubated at 35 ± 2 °C for 3 days or at 28 ± 2 °C for 7 days, respectively. This methodology was designed based on specifications of the European Pharmacopeia monographs (2.6.12. Microbiological examination of non-sterile products: total viable aerobic count).

### In vitro release

The in vitro release of TH from TH-NP against free TH was carried out using vertical diffusion Franz cells (FDC-400, Vidra-Foc, Barcelona, Spain) with a thermal bath set to 32 °C, to mimic skin in vivo conditions, under constant stirring. For this study, methylcellulose membranes (Dialysis Tubing – ViskingCode DTV12000.03.000, Size 3, Inf Day 20/32″–15.9 mm, MWCO–12–14.000 Da, Liverpool Road, London, UK) were placed between donor/receptor compartments (2.54 cm^2^). Samples of TH or TH-NP were added to the donor phase (0.5 mL) and the receptor phase was filled with Transcutol P®:water (50:50), maintaining sink conditions. Aliquots of 300 μL were collected at selected times, replaced with the same amount of receptor medium [33]. Data were analyzed by HPLC and processed with the Boltzmann Sigmoidal mathematical model, Eq. (2), using GraphPad®.

2 $$Y=\frac{Bottom+(Top-Bottom)}{(1+\mathrm{exp}(\frac{\left(V50-X\right)}{Slope})}$$

### Ex vivo skin permeation

Ex vivo human skin permeation was carried out by vertical diffusion Franz Cells, using the same methodology as described above. Human skin was obtained from abdominal plastic surgery (Hospital de Barcelona, SCIAS, Barcelona, Spain), following a protocol approved by the Bioethics Committee of the Barcelona-SCIAS Hospital. Skin samples (2.54 cm^2^, 0.4 mm thick) were clamped into the Franz cells with the SC facing up [50]. Previously, some of the skin samples were scratched with sandpaper to mimic damaged skin SC. The donor compartment was filled (0.5 mL) with TH or TH-NP (0.25%). Data were analyzed by HPLC and processed using GraphPad®. The skin permeation parameters were calculated by Eq. (3):

3 $$J=Kp \cdot C_0$$

where *J* is the flux, *Kp* is the permeability coefficient and *C*_*0*_ is the initial concentration of the active [51].

The amount retained inside the skin was assessed by tape striping and skin extraction techniques. Firstly, skin samples were washed with sodium lauryl sulphate (0.02%) and rinsed with distilled water, dried, cut, and weighted. For determination of the amount retained in the SC, tape stripping assay was developed based on previous authors with minimal modifications [52]. The first layers of the skin were removed by 7 strips of the same region of the SC using a transparent label dressing (3 M Tegaderm®, 6 × 7 cm, 10u, Spain, S.A.). The strips were added into 4 mL of ethanol and placed into an ultrasonic bath (JP, Selecta) for 20 min for compound extraction. For determination of the total amount retained inside the deeper layers, the rest of the skin was perforated, added into 2 mL of ethanol:water (50:50) and then kept in the ultrasonic bath for 20 min [51]. The amount of thymol extracted was determined by HPLC and calculated using the recovery factor previously obtained.

To determine the permeation route of TH-NP, vertical diffusion Franz cells were used as described before. Ex vivo pig skin penetration was obtained from the animal house (Bellvitge, University of Barcelona), used in accordance with the protocol approved by the Ethics Committee of the University of Barcelona. For this study, rhodamine-labelled PLGA (R-PLGA) was synthesized as previously described [43]. R-PLGA was used at 0.01% into TH-NP, added in the organic phase with PLGA. R-TH-NP were applied onto the ex vivo pig skin 0.64 cm^2^ (donor compartment) and allowed penetration for 24 h. Skin samples were washed, fixed in PBS containing 4% paraformaldehyde (PFA) for 4 h, followed by cryoprotection into PBS with 30% sucrose for 24 h, snap-frozen in isopentane at − 50 °C, then kept overnight at − 80 °C. Samples were mounted in O.C.T.® Compound (Tissue-Tek®, Sakura) and sliced with cryostat microtome (LEICA CM3050 S) at − 20 °C onto glass-slides Superfrost® Plus (Menzel-Glaser, Thermo Scientific, USA), covered with Fluoromount G® (Invitrogen, Thermo Fisher Scientific, USA). Samples were visualized by confocal laser scanning microscopy (Zeiss LSM 880), using objective lens 10× 0.45. Images were acquired using Zen Black 2.3 software performing z-stack sections and thus processed with ImageJ software.

### Cytotoxicity studies in HaCaT cells

Human keratinocytes (HaCaT) cells were cultured in high glucose DMEM (Dulbecco's Modified Eagle's Medium (Thermofisher), supplemented with 10% fetal bovine serum (FBS), 2 mM l-glutamine, 100 units/mL penicillin G and 100 µg/mL streptomycin. Cells were incubated at 37 °C and 5% CO_2_ and experiments were performed when cells reached 80–90% of confluence.

Cytotoxicity of TH-NP was determined by MTT (3-(4,5-Dimethylthiazol-2-yl)-2,5-diphenyl tetrazolium bromide) assay, by reduction of tetrazolium salt by intracellular dehydrogenases of viable living cells. TH-NP were tested at concentrations up to 1 mg/mL. The assay was also performed with TH-NP previously washed thrice (TH-NP-w) to remove excess of free TW (centrifugation at 14,000 rpm for 15 min). Briefly, HaCaT cells were seeded in 96-well plates with 100 μL of culture medium (DMEM) at a density of 2 × 10^5^ cells/well, adjusted in automated cell counter (Countess, Invitrogen, Thermofisher). Cells were incubated with samples for 24 h. Then, the medium was removed and MTT (Sigma-Aldrich Chemical Co, St. Louis, MO, USA) was added at 0.25% in PBS. After 2 h incubation, the medium was replaced by 100 µL DMSO (99% dimethyl sulfoxide, Sigma-Aldrich) [53]. Cell viability was then measured at wavelength of 570 nm in a Modulus® Microplate Photometer (Turner BioSystems Inc., Sunnyvale, CA, USA). Results were expressed as percentage of cell survival relative to untreated cells.

### Cellular uptake of TH-NP

Cellular uptake of TH-NPs was assayed in HaCaT cells seeded in an 8-well µ-slide (Ibidi®) following the same methodology as described before. Cells were incubated with or without R-TH-NP for 2 h at the indicated concentration, using FBS/phenol red free medium. Cell membranes were stained with wheat germ agglutinin (WGA) Alexa-488 (Molecular Probes) at 1 µg/mL for 15 min followed by fixation with paraformaldehyde 3% for 25 min. Cell nuclei were stained with 4′,6-diamidino-2-phenylindole (DAPI, Sigma Aldrich, Spain) at 0.5 µg/mL for 15 min. Internalization of NPs in HaCaT cells was assessed by confocal microscopy (Leica TCS SPII), 63× oil immersion objective lens [43]. Images were processed using Fiji image software.

### In vitro antioxidant efficacy in HaCaT cells

The antioxidant activity of TH, TH-NP, and B-NP (blank NPs) was assayed in HaCaT cells by quantification of ROS using the fluorogenic probe H2DCFDA. Cells were seeded in 96-well plates at 2 × 10^5^ cells/well (100 µL) for 72 h. Cells were loaded with the fluorogenic dye H_2_DCFDA (2′,7′-dichlorodihydrofluorescein diacetate) at 25 µM diluted in DMEM medium absent of phenol red and FBS, for 45 min in the dark. This fluorogenic dye passively diffuses into the cells, being deacetylated by intracellular esterase and emits fluorescence upon oxidation by reactive oxygen species (ROS) [54]. Then, cells were washed with PBS and incubated for 2 h with TH, TH-NP or B-NP. After this period, 10 µL of H_2_O_2_ 20 mM was added to each well. Untreated cells with or without H_2_O_2_ were used as positive and negative controls, respectively. Fluorescence was measured at excitation and emission wavelengths of 485 and 530 nm, respectively. Data were acquired at times t0 up to 120 min. Data of the positive control (H_2_O_2_) at 2 h, were used to normalize values (%). Background fluorescence of the negative control was subtracted from all measurements.

### In vitro antimicrobial efficacy

*S. epidermidis* was grown overnight 37 °C in Mueller Hinton Broth (MHB) culture medium (Oxoid, Basingstoke, UK). Prior to each experiment, the inoculum was prepared in PBS adjusted to 0.5 MacFarland (McF) standard, to obtain a suspension with a cell density at the range of 1.5 × 10^8^ colony forming units/mL (CFU/mL). Microbial count of *S. epidermis* was performed in TSA plates, incubated at 37 °C. The *C. acnes* was cultured in Brain Heart Infusion (BHI) medium (Oxoid, Basingstoke, UK) for 48 h at 37 °C under anaerobic conditions using parches (AnaeroGen®, Oxoid, Basingstoke, UK) and indicator (Oxoid, Basingstoke, UK). Prior to each experiment, the inoculum was prepared in PBS adjusted to 0.5 McF. Microbial count of *C. acnes* was performed in clostridium reinforced medium (CRM) plates, as recommended for anaerobia growth, incubated at 37 °C.

The MIC of TH and TH-NP were determined using the broth microdilution assay [55]. Briefly, double concentrated sample dilutions were prepared and added (100 μL) to double concentrated culture medium (100 μL) in a 96-well polypropylene microtiter plate (Costar, Corning Incorporated, Corning, USA). Inocula were prepared to yield a final concentration of 5 × 10^5^ CFU/mL. For *S. epidermidis,* 10 µL were transferred to inoculate wells with final TH concentrations of 2 to 1024 µg/mL, followed by incubation at 37 °C for 18 to 20 h. For *C. acnes,* 20 µL was used to inoculate wells with concentrations ranging from 2 to 1000 µg/mL and the plate was incubated at 37 °C for 48 h under anaerobiosis. Thus, the MBC was performed by transferring 10 µL of each sample presenting no visible growth of *C. acnes* into BHI plates. These were further incubated as described before. Growth controls were used for the above experiments: presenting antimicrobial sterility (negative) and absent of antimicrobial (positive). Clindamycin was also used as an active control for both microorganisms.

The determination of the decimal reduction time assay, explores the antimicrobial activity of TH and its derivative TH-NP on reducing bacteria viability at determined contact times [56]. For *C. acnes*, formulations were diluted with water up to 250, 500 and 1000 µg/mL representing the MIC, 2 × MIC and 4 × MIC, respectively. For *S. epidermidis*, formulations were used at 1000 µg/mL, representing twice as MIC. Inocula were prepared in PBS at 10^8^ CFU/mL and used to inoculate (100 µL) each experimental sample of 10 mL, incubated at 32 °C. The determined times were 0, 1, 2, 3 and 6 h or 0, 3, 8, 24 and 48 h for *C. acnes* and *S. epidermidis*, respectively. After incubations of each time set, an aliquot of 1 mL of each sample was neutralized in 9 mL of Berens diluent (Scharlab, Barcelona, Spain) for 15 min, then, diluted in PBS on subsequent 10-folds. Drop count method (10 µL) was performed in CRM and TSA agar plates, *for C. acnes* and *S. epidermidis*, respectively, incubated at 37 °C as described previously. Bacterial viability was expressed as CFU/mL against time (h). The decimal reduction time, the time taken to reduce by 10% the initial log_10_CFU, was determined calculating the inverse of the slope (1/b).

The antimicrobial activity was also evaluated by SEM. For this, *C. acnes* was cultured for 48 h in BHI culture media in an incubator shaker (Innova® 4080, New Brunswick Scientific) at 37 °C under anaerobic conditions. The concentrated inoculum was transferred (900 µL) to each tube containing 100 µL of TH or TH-NP at 0.1% or sterile distilled water (control) and incubated in the shaker for 1 h. After samples were centrifuged (10,000*g* for 5 min), supernatants were discarded and the concentrated pellets were placed into poly-l-lysine coated coverslips and kept at room temperature for 24 h [57]. Samples were fixed for 4 h with phosphate buffer 0.1 M pH 7.4, containing 4% paraformaldehyde and 2.5% glutaraldehyde, then post-fixed with 1% osmium tetroxide (with potassium ferrocyanide) for 1 h, at 4 °C. After dehydration with alcohol gradients, samples were dried at critical point (Emitech K850), mounted on a conductor adhesive disc (Carbon tabs, Agar Scientific), followed by carbon coating under evaporation (Emitech 950). Images were analyzed by SEM (scanning electron microscopy, Jeol JSM-7001F).

### Ex vivo antimicrobial efficacy

The bacterial viability was evaluated on treated human skin explants obtained from abdominal plastic surgery (Hospital de Barcelona, SCIAS, Barcelona, Spain), based on other researcher protocols with modifications [58]. Skin samples were cut with a cryostat (Leica Microsystems, Wetziar, Germany) in 0.6 cm^2^, washed with ethanol followed by sterile PBS, for 2 and 10 s, respectively, to remove possible existing bacteria. Once dried with sterile filter paper, skins were placed into petri dishes (purchased from Fischer Scientific) with the SC facing up, onto PBS-wet sterile filter paper to keep dermis moisture. Two experiments (prevention and treatment) were set up for 24 h incubation at 32 °C, in the presence of humidity. A fresh overnight culture of *C. acnes* was suspended in PBS (1.5 × 10^8^ CFU/mL) and skin samples were inoculated with 10 µL. For the pre-treatment study, TH-NP or TH were applied on skin samples (30 µL) and incubated at 32 °C for 8 h, followed by inoculation with *C. acnes* (30 µL) for 16 h. For the post-treatment study, skin was first inoculated for 30 min and then treated with products for 24 h. At the end of the experiment, skin samples were neutralized in 1 mL Berens diluent (Scharlab, Barcelona, Spain) for neutralization (15 min) followed by extraction for 10 min using a sonication bath (JP, Selecta, Spain). The extraction method was previously optimized by testing the control at two extraction times (3 to 15 min), controlling bacteria viability by sonication process. Positive controls were also performed using PBS. Tenfold dilutions were performed and 100 µL of each sample was spread individually onto CRM agar plates and incubated under anaerobiosis at 37 °C for 48 h. Viable bacteria count was expressed as log/CFU per treated skin.

The analysis of bacterial viability on dose-dependent study on treated skin was also performed using the same technique as described above, with further modifications [58]. A fresh overnight culture of *C. acnes* was prepared in PBS and skin samples were inoculated (10 µL). After 30 min, 30 µL of TH or TH-NP were administered as a single or repeated dose (1, 2 or 3), at preselected times (0, 12 and 18 h), completing a total incubation of 24 h at 32 °C, in the presence of humidity. Then, skin samples were neutralized and extracted as described above. These were tenfold diluted and transferred to CRM plates by drop-count method (10 µL). Plates were incubated under anaerobic conditions at 37 °C for 48 h. Viable bacteria were expressed as CFU per treated skin (Additional file 1).

A simulation of skin infection was performed in fresh human skin explant (Hospital de Barcelona, SCIAS, Barcelona, Spain) and analyzed by transmission electron microscopy. The fat tissue of skin samples, obtained from human abdominal plastic surgery, was removed manually with sterile surgical razors. Skin samples were cut and placed on a 0.64 cm^2^ Franz diffusion cell. The receptor compartment was filled with PBS, and the skin was inoculated with 20 µL of *C. acnes* (10^8^ CFU/mL) and incubated for 16 h at 32 °C, followed by treatment with TH or TH-NP (100 µL) for 8 h incubation. For electron microscopy, skin samples were fixed for 2 h with 4% paraformaldehyde and 2.5% glutaraldehyde in 0.1 M sodium cacodylate buffer (pH 7.4), postfixed with 1% osmium tetroxide for 2 h at 4 °C (all from Sigma Aldrich), stained in 0.5% uranyl acetate (from Fischer Scientific) for 45 min at 4 °C and finally, dehydrated gradually in 30 to 100% ethanol [40]. Samples were infiltered in EPON resin [Eponate 12 (23.5 g), dodecenyl succinic anhydride DDSA (12.5 g) and Methyl nadic anhydride MNA (14 g)] (from Sigma Aldrich). Inclusions were performed gradually diluted in ethanol and finally for 3 h using a catalyst [DMP-30 (2,4,6-tris(dimethylaminomethyl)phenol), 0.37 g] (purchased from Sigma Aldrich). Polymerization was carried out for 48 h at 60 °C. Blocks were sliced in thin sections with Ultracut microtome (LEICA), further fixed on copper grids and stained with uranyl acetate 2% for 10 min. Analysis was performed by TEM and images were obtained with Megaview III.

## Supplementary Information

**Additional file 1: Figure S1.** Morphology of TH-NP by TEM. (A) 1 month at 4 °C (B) 1 month at 25 °C and (C) 12 months at 4 °C. Arrows indicate aggregation. Scale bar: 200 nm. **Figure S2.** Stability behavior of TH-NP plotted as light backscattering (%) vs sample height, at several storage conditions: (A) 4 °C up to 12 m, (B) 25 °C and (C) 37 °C up to 3 months. The scans are shown from the bottom to the top of the vial from the left to the right, as mean values of hourly measurements for 24 h. **Figure S3.** Morphology of *C. acnes* observed by TEM after negative staining. Scale bar 500 nm.

## Data Availability

Not applicable.
