# Supplementary material for: Thymol-loaded PLGA nanoparticles: an efficient approach for acne treatment
Source: J Nanobiotechnology. 2021 Nov 8;19:359. doi: 10.1186/s12951-021-01092-z (PMC8577023; doi:10.1186/s12951-021-01092-z)
Supplement: Supplementary file 1 — Additional file 1: Figure S1. Morphology of TH-NP by TEM. (A) 1 month at 4 °C (B) 1 month at 25 °C and (C) 12 months at 4 °C. Arrows indicate aggregation. Scale bar: 200 nm. Figure S2. Stability behavior of TH-NP plotted as light backscattering (%) vs sample height, at several storage conditions: (A) 4 °C up to 12 m, (B) 25 °C and (C) 37 °C up to 3 months. The scans are shown from the bottom to the top of the vial from the left to the right, as mean values of hourly measurements for 24 h. Figure S3. Morphology of C. acnes observed by TEM after negative staining. Scale bar 500 nm. [file 12951_2021_1092_MOESM1_ESM.pdf]

Additional file 1

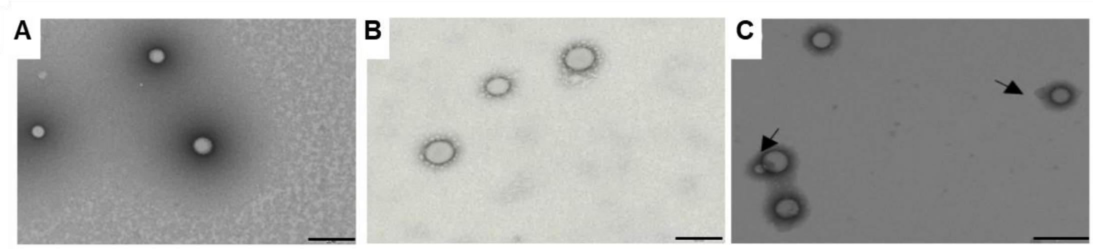

Figure S1. Morphology of TH-NP by TEM. **(A)** 1 month at 4 °C **(B)** 1 month at 25 °C and **(C)** 12 months at 4 °C. Arrows indicate aggregation. Scale bar: 200 nm.

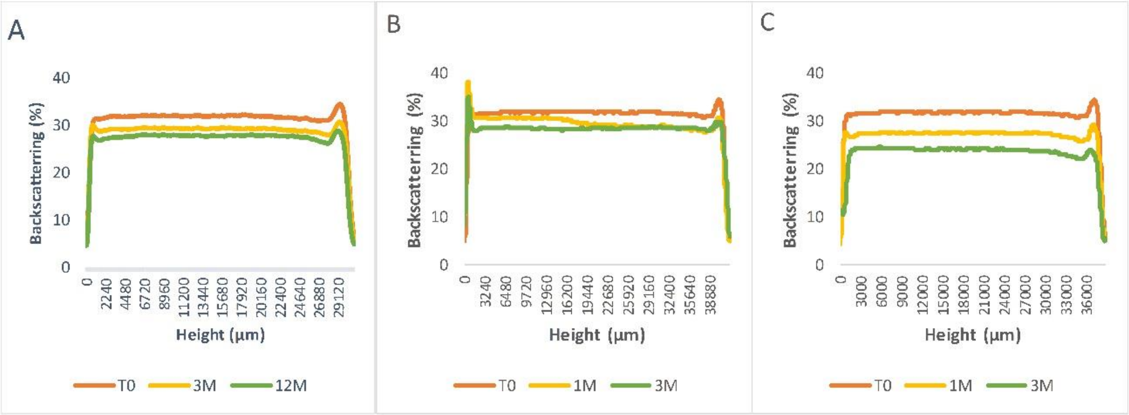

Figure S2. Stability behavior of TH-NP plotted as light backscattering (%) vs sample height, at several storage conditions: (A) 4 °C up to 12 m, (B) 25 °C and (C) 37 °C up to 3 months. The scans are shown from the bottom to the top of the vial from the left to the right, as mean values of hourly measurements for 24 h.

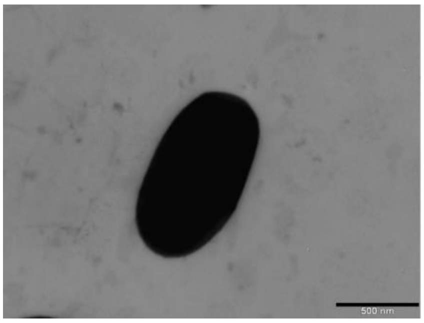

Figure S3. Morphology of *C. acnes* observed by TEM after negative staining. Scale bar 500 nm.
